# Selective infarct zone imaging with intravenous acoustically activated droplets

**DOI:** 10.1371/journal.pone.0207486

**Published:** 2018-12-14

**Authors:** Songita A. Choudhury, Feng Xie, Shelby Kutty, John Lof, Elizabeth Stolze, Thomas R. Porter

**Affiliations:** University of Nebraska Medical Center, Omaha, NE, United States of America; Monash University, AUSTRALIA

## Abstract

**Background:**

Microbubbles (MB) can be compressed to nanometer-sized droplets and reactivated with diagnostic ultrasound; these reactivated MB possess unique imaging characteristics.

**Objective:**

We hypothesized that droplets formed from compressing Definity MB may be used for infarct-enhancement imaging.

**Methods:**

Fourteen rats underwent ligation of their left anterior descending (LAD) artery, and five pigs underwent 90 minute balloon occlusions of their mid LAD. At 48 hours in rats, transthoracic ultrasound was performed at two and four minutes following 200 μL intravenous injections (IVI) of Definity droplets (DD), at which point the MI was increased from 0.5 to 1.5 to assess for a transient contrast enhancement zone (TEZ) within akinetic segments. In pigs, 1.0 mL injections of DD were administered and low frame rate (triggered end systolic or 10 Hz) imaging 2–4 minutes post iVI to selectively activate and image the infarct zone (IZ). Infarct size was defined by delayed enhancement magnetic resonance imaging (DE-MRI) and post-mortem staining (TTC).

**Results:**

Increasing MI to 1.5 (at two or four minutes after IVI) resulted in a TEZ in rats, which correlated with infarct size (r = 0.94, p<0.001). A TEZ was not seen at 2–4 minutes in any rat (n = 8) following Definity MB injections. Fluorescent staining confirmed DD presence within the infarct zone 10 minutes after intravenous injection. In pigs, selective enhancement within the IZ was achieved by using a low frame rate single pulse harmonic mode; IZ size matched the location seen with DE-MRI and correlated with TTC defect size (r = 0.90, p<0.05).

**Conclusion:**

DD formulated from commercially available MB can be acoustically activated for selective infarct enhancement imaging.

## Introduction

The perfluorocarbons that are utilized for commercially available microbubble preparations can be condensed into a liquid phase and remain in this state even at room temperatures [1–4]. The fluorocarbon perfluoropropane (C_3_F_8_) gas that is used for a lipid encapsulated microbubble [5] has a unique property of condensing to droplet form under cooled pressure and maintaining this superheated liquid state even at body temperatures. The condensed droplets formed from cooled compression are typically 200–330 nanometers in size, which is what would be predicted from ideal gas laws for 1–2 micron microbubbles [6], and remain in this condensed phase even following intravenous injection in large animals [7, 8]. One potential advantage of this size is that they could cross defective endothelial cell junctions that exist both following acute myocardial ischemia and ischemia-reperfusion [9]. Alternatively, these droplets may have altered surface charge and surface area properties that result in increased attachment to leukocytes slowly rolling across the defective venular endothelial surface following ischemia-reperfusion [10]. Therefore, they may accumulate within the infarct zone where, at some time point following venous injection, they could be activated and imaged again with diagnostic ultrasound. In order to image these droplets after they have reached their desired location, they must be vaporized. The peak negative pressure threshold required for acoustic droplet vaporization has been shown to be frequency dependent, with higher frequencies having lower thresholds [2, 4]. Therefore, current methods of vaporization have employed “activate high frequencies, listen low frequencies” in order to image the droplets [11–13]. These “activate high frequencies” are in the 8 MHz range, and are well beyond what would be expected to be clinically feasible for transthoracic imaging of the myocardial microcirculation in large animals and humans due to attenuation from lung and intervening intracardiac chambers.

We have demonstrated with <2 MHz frequencies, that diagnostic transthoracic ultrasound pressures at or above 1.0 mechanical index (MI) can reactivate these droplets [7, 8]. Since droplet velocity (whether intra or extravascular) within the infarct zone would be expected to be relatively stationary with respect to normal microcirculation and left ventricular cavity, selective activation and imaging of droplets within the infarct zone may be possible. This may result in an infarct enhancement zone similar to what is seen with delayed enhancement magnetic resonance imaging. In this study, we hypothesized that a) intravenously injected perfluoropropane Definity droplets would accumulate within an infarct zone, and b) acoustic activation and imaging with diagnostic ultrasound would detect this zone and correlate with infarct size.

## Methods

### Microbubble and nanodroplet formulation

Definity (Lantheus Medical, N. Billerica, MA) was used in all the studies. Definity microbubbles (DMB) were activated via mechanical agitation (Vialmix shaker, Bristol-Myers-Squibb, New York, NY) for 45 seconds [5]. Definity droplets (DD) were formulated according to a previously described protocol [7, 8]. Briefly, 2 mL vials of DMB were agitated for 45 seconds, the DMB solution was diluted two-fold with normal saline and submerged in a 70% isopropyl alcohol bath at a temperature between -10°C and -15°C for 3 minutes. The DMB 10% solution was removed from the ice bath and the syringe placed into a trigger clamp (DeWalt, Baltimore, Maryland) for compression into DD. Pressure was applied for 3 minutes until the solution cleared, indicating condensation. Although we did not monitor the pressure with every formulation, we have previously determined with a high pressure inflation device (Merit Medical Systems, Jordan, UT) that 9–12 atmospheres of pressure are required for condensation at this temperature.

The solution was then allowed to warm to room temperature before administration. DMB and DD were both sized (n = 3 for each solution) using a Nano Zetasizer (Malvern Nano ZS, Malvern Instruments Ltd., Malvern, Worcestershire, U.K.), which is capable of sizing particles from 50 nm up to 10 μm diameter. The Zetasizer utilizes dynamic light scattering to determine particle size. Microbubble and droplet concentrations were determined with Nanosight laser scattering analysis (Malvern, Worcestershire, UK; Malvern.com).

### Experimental setup

**Fig 1:**
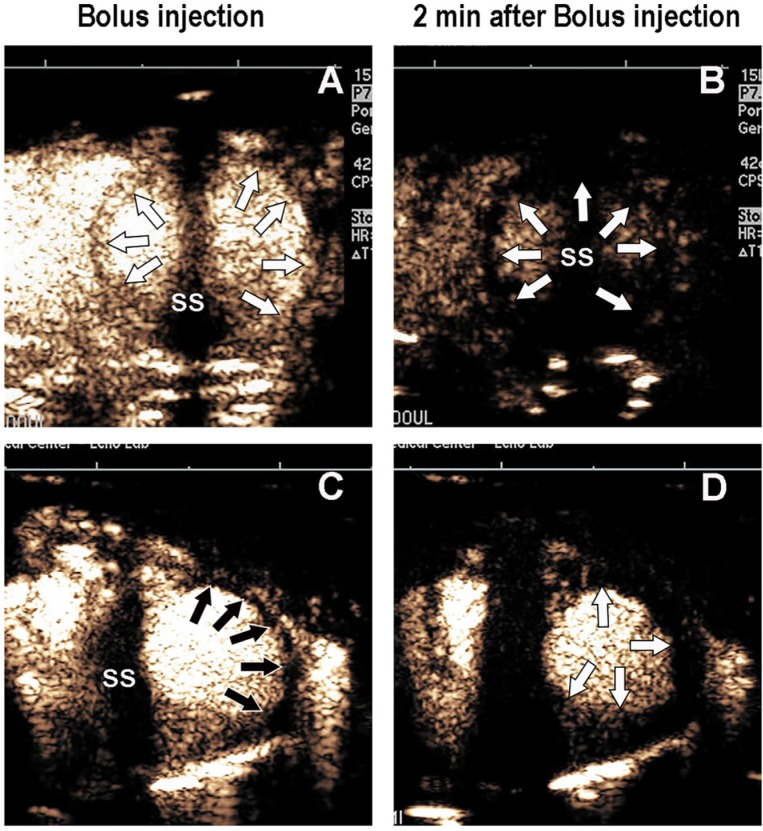
A bolus intravenous injection of Definity MB at 0.5 MI in a rat without infarction immediately following injection (Panel A) and at two minutes following injection (Panel B), where myocardial contrast is no longer present when using a triggered end systolic frame rate. In a rat with LAD infarction, the bolus microbubble injection demonstrated an anterior/anterolateral segment contrast defect (Panel C) immediately following injection but no myocardial contrast in normal or abnormal segments at two minutes (Panel D). SS = sternal shadow. White arrows = normal myocardium. Black arrows = contrast defects.

The Institutional Animal Care and Use Committee at the University of Nebraska Medical Center approved all rodent and porcine studies. All animals were group housed. Fourteen rats (Sprague-Dawley, Charles River, Portland, MI) underwent left anterior descending (LAD) artery ligation using an established protocol [14] under isoflurane anesthesia (2%) and with aseptic technique. Analgesia (buprenorphine, 0.05 mg/kg, SC) was administered prior to surgery and for two days post operatively (each 8–12 hrs). Following confirmation of a persistent occlusion, the chest incision was closed and the animals recovered. At 48 hours post ligation, the animals were re-anesthetized (isoflurane, 2%), and a Siemens 15L8 transducer (Siemens Acuson Sequoia, 7-MHz fundamental frequency, Siemens Healthcare, Mountain View, CA) was used to perform transthoracic imaging. Once a short axis mid papillary muscle image was obtained, the transducer was placed in a fixed position over the chest wall using a cross clamp and stabilized with a ring stand. Baseline two-dimensional images were captured to assess regional wall motion. For droplet detection in rats, the imaging modality was contrast pulse sequencing (CPS; Siemens Healthcare), which is a multi-pulse sequence scheme that sends pulses of alternating amplitude and polarity. A 200 μL intravenous injection (IVI) of a 1:2 dilution of DMB was first administered to the animals during low MI (0.5 MI) imaging (triggered at 1 frame every 3–4 cardiac cycles). Contrast enhancement within the cavity or myocardium at this MI setting following the bolus injection was recorded as “present or absent”, and contrast defects that were visualized were planimetered off line [Fig 1]. In eight of the rats at two minutes following IVI, any residual myocardial or cavity contrast was noted at the 0.5 MI setting, following which the MI was transiently increased to 1.5 for a period of five seconds to examine for any changes in contrast intensity in the infarct or remote normal myocardial zones. The intravenous line was flushed at 10 minutes following the last DMB injection to remove any residual microbubbles in the line.

**Fig 2:**
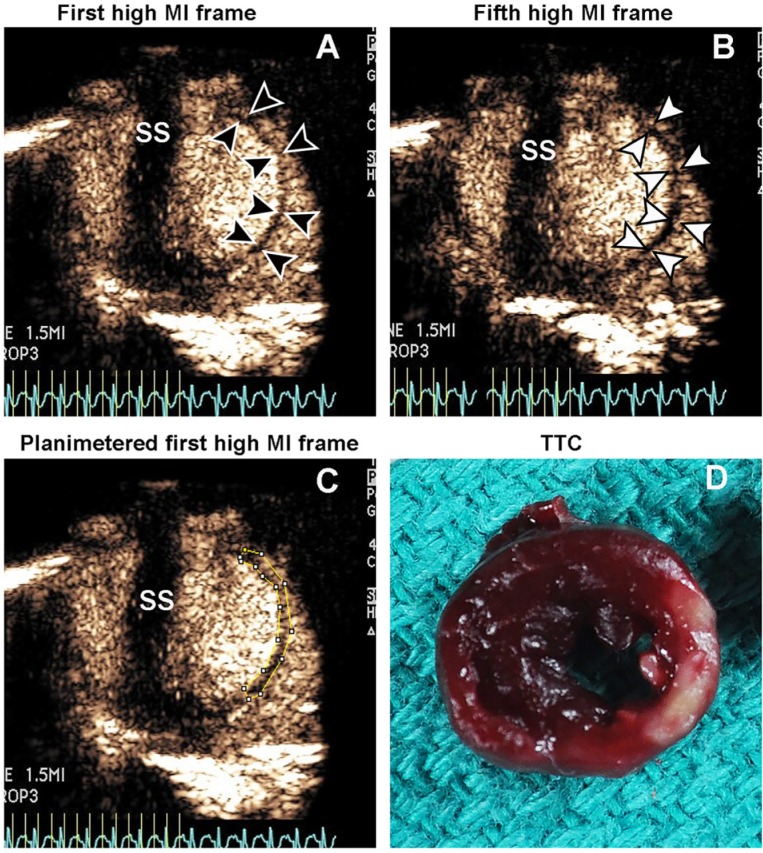
Two minutes post intravenous injection of definity droplets, myocardial and cavity enhancement are observed when the MI is increased to 1.5 (Panel A). After five frames at this high MI, a decrease in contrast is observed in the transient enhancement zone (TEZ), with endocardial and epicardial borders delineated in Panels A and B. The size of the TEZ is planimetered in Panel C, and the corresponding infarct zone is demonstrated by TTC staining in Panel D. IVI = intravenous injection; SS = sternal shadow.

**Fig 3:**
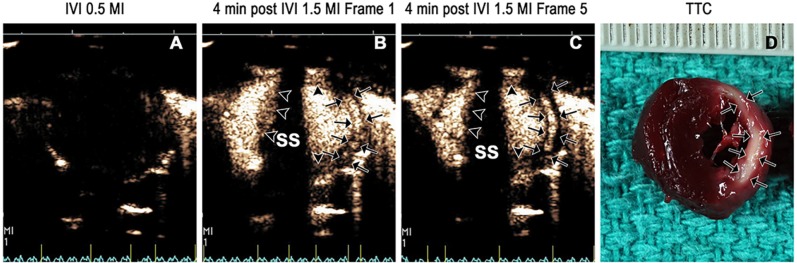
At 4 minutes post intravenous injection of definity droplets, there is no contrast at 0.5 MI (Panel A), but at 1.5 MI there is transient enhancement within the infarct zone (arrows; Panel B) which disappears in all but the central portion of the infarct zone with repeated high MI imaging impulses (arrows; Panel C), while the normal myocardium enhances as a result of the bolus of microbubbles produced by acoustic activation within the left ventricular cavity (arrowheads in Panels B and C). The corresponding TTC image is shown (Panel D). IVI = intravenous injection; SS = sternal shadow.

Subsequent to this, a 200 μL intravenous injection of a 1:1 dilution of DD was given. Any contrast produced by the 0.5 MI setting was noted, which was consistently no or faint left ventricular cavity contrast. At two to six minutes post IVI, the MI was transiently increased to 1.5 (while triggering at end systole once every three to four cardiac cycles) which resulted in significant left ventricular and myocardial enhancement from DD activation, including transient enhancement (3–4 frames) within the hypokinetic infarct zone (termed TEZ). The TEZ was defined then as the planimetered area of transient myocardial contrast enhancement observed within the hypokinetic zone as the MI was increased to 1.5, and which partially or completely disappeared on subsequent triggered frames at the high MI setting (see examples in Figs 2 and 3). With CPS even at low frame rates, the non-infarct zones outside the TEZ and left ventricular cavity typically exhibited persistent homogenous myocardial contrast enhancement in this same time period.

After the DMB and DD injections, a 200 μL injection of fluorophore labeled DD (formulated as described below) was administered without imaging, and the animals were euthanized 10 minutes later. The location of the transducer probe on the chest wall was marked to ensure the same location was sliced for subsequent post mortem staining and fluorescent microscopy. Triphenyltetrazolium chloride (TTC) staining for infarct size was performed at 37°C using standardized techniques [15].

### Modification of pulse sequence scheme selectively enhance the infarct zone in a large animal model

In five otherwise normal swine, balloon occlusion of the mid left anterior descending was performed for 90 minutes followed by reperfusion and recovery from anesthesia. Procedures were performed under isoflurane anesthesia (1.5–4%) utilizing aseptic technique. Analgesia was provided by carprofen (2–4 mg/kg, SC). At 48 hours post reperfusion, the pigs underwent cardiac magnetic resonance imaging (CMRI) using a 1.5 Tesla field strength magnet (Philips Achieva) again utilizing isoflurane inhalation anesthesia. Imaging sequences included standard steady state free precession imaging (TR 3.0 msec, TE 1.3 msec) followed by T2 weighted spin echo images in the same short axis views. Delayed enhancement imaging (DE-MRI) was then performed in the same short axis offsets at 10 minutes following a 0.2 mmol/kg injection of gadopentetate dimeglumine (Magnevist; Bayer).

Following this, the pigs were removed from the magnet and proceeded to echocardiographic imaging. Prior to DD injection, short axis ultrasound images were obtained to correspond to a region with a resting wall motion abnormality and a scar zone by DE-MRI. Immediately following this, 1.0 ml injections of 1:1 dilutions of DD were administered. Based on the rat studies that used multipulse schemes (amplitude and phase modulating) that resulted in simultaneous activation and imaging of droplets within the cavity and normal microcirculation, we hypothesized that single pulse harmonic or ultraharmonic imaging at slower frame rates (or triggered frame rates) would permit activation *and* subsequent imaging of only the slow moving or stationary droplets that were retained within the infarct zone. Therefore, for these studies we used a Philips iE 33 (Philips Healthcare; Andover, MA) phased array transducer to activate and image at two to four minutes post IVI. Harmonic imaging was performed using either a 1.3/3.4 MHz ultraharmonic or 1.7/3.4 MHz harmonic setting at low frame rates (10 Hz or end systolic triggered) with MI’s of either 0.7, 1.0 or 1.3. All images were digitally stored. Pigs were then sacrificed and post-mortem TTC staining utilized again to define the extent of the infarct zone in the same short axis plan used to detect for acoustic activation and imaging.

### Fluorescent droplets

In order to confirm presence of DD within the infarct zone in the rat studies, Definity microbubbles were fluorescently labeled with a commercially available lipophilic fluorophore, dioctadecyltetramethylindocarbocyanine (DiI), prior to droplet formulation. DiI (2 μL/vial) was added to preformed DMB as previously described [16]. This solution was then emulsified using a Vialmix as described previously. The droplets were formulated using the same compression technique described for non-fluorophore containing droplets. A 200 μL (rats) or 1 milliliter (pigs) bolus injection of the fluorophore containing droplets was administered to the animal after completion of all imaging protocols. Animals were euthanized 10 minutes following injection. Post mortem 1 mm thick short axis sections of the myocardium corresponding to the location used for short axis imaging and for TTC staining were collected.

### Image analysis

In rats, quantitative measurements of the TEZ following DD injections, and contrast defects produced by DMB, were determined by a consensus of two experienced reviewers (FX and TRP) blinded to the TTC and fluorescent images. The triggered images were digitally reviewed during the transition from 0.5 to 1.5 MI, which resulted in immediate left ventricular cavity and normal myocardial zone enhancement and a transient (3–4 triggered frames) enhancement within the akinetic zone detected on short axis two-dimensional echocardiographic images. The normal myocardium was distinguished from the TEZ by the persistence of myocardial contrast beyond the transient enhancement. The TEZ was planimetered with Image J (https://imagej.nih.gov/ij/) software by analyzing size by side the TEZ during and following its enhancement period, as shown in Fig 2. In pigs, any selectively enhanced zone that was seen at the 1.0 or 1.3 MI while in low frame rate imaging was planimetered.

Fluorescent microscopy (Carl Zeiss Canada, Toronto, ON) was performed on post mortem sections to identify any locations exhibiting emission spectra near 590 nm (emission spectra for DiI). The Image J software was used to also quantify the fluorescent zone and unstained TTC area.

### Statistical comparisons

Statistical analysis was performed using SigmaPlot V13.0 (Systat Software, Inc., San Jose, CA). Since we were comparing continuous variables, the size of the TEZ was compared to three other modes of infarct size detection using a Pearson correlation coefficient. The first was the planimetered size of the contrast defect obtained following DMB injections, which was chosen from triggered frames within the two minutes following the DMB injections. The second comparison was with the size of the infarct zone on post mortem TTC staining. The final comparison was with the size of fluorescently DiI stained area on the same short axis slice used for TTC staining. Linear regression was used to compare TTC unstained defect size and fluorescent area with both the contrast defect observed following DMB injections and the TEZ observed with droplet activation at >2 minutes following DD injection. In six rats, a repeat 200 uL injection of DD was administered to determine reproducibility of the planimetered TEZ size using Bland-Altman analysis. In pigs, correlation coefficients were used to compare the selectively enhanced area at high MI low frame rate imaging and the infarct zone by TTC.

## Results

### Microbubble and nanodroplet sizing/Concentrations

**Fig 4:**
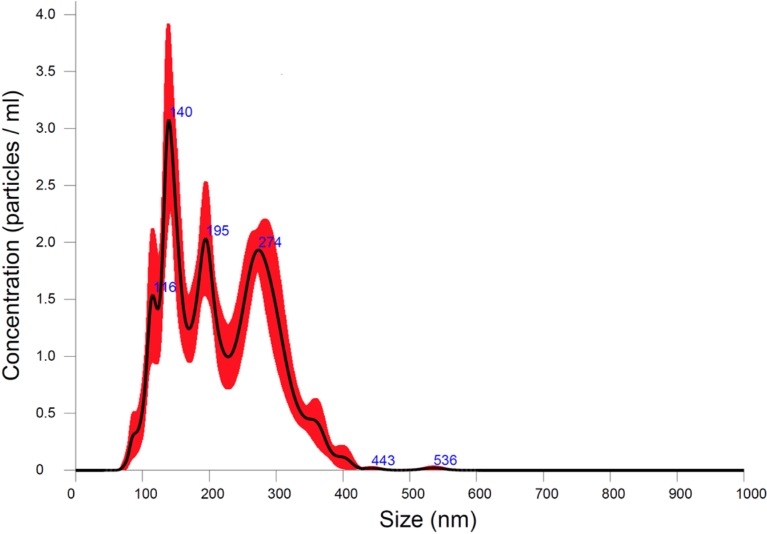
The size distribution of definity droplets formulated with the cooling and compression techniques described in the methods section. The shaded areas in the plots represent the range of values obtained in the five samples tested.

The formulated DD had a polydisperse size distribution, with two main peak sizes of 82 nm and 427 nm (Fig 4).

The DMB were monodisperse with a mean size of 767 nm. The number of droplets in the 200 μL doses used for rat studies was 1.7x10^12^, while the concentration of DMB in the 200 μL solution was 4.9x10^11^. Note these are larger numbers than recorded in the package insert [5] because of the larger size range of measured microbubbles and droplets that could be sized with the Zetasizer. Droplet doses tested in pigs were 8.5 x 10^12^ droplets in the one-milliliter bolus injections.

### Infarct zone detection with different pulse sequence schemes

No changes in heart rate (mean heart rate 304± 95 beats per minute before injection, 291 ± 31 beats per minute after injection), respiratory rate, or oxygen saturation were seen following DD or DMB injections in any rat. By TTC staining, infarct size ranged from 0–11.6 mm^2^ of the mid to distal papillary muscle short axis plane imaged. Of the 14 animals, 11 had transmural infarctions and 3 had either no or a non-transmural infarction (S1 Table).

**Fig 5:**
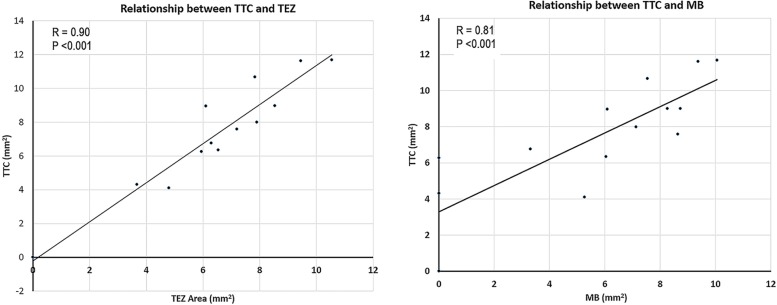
Correlation between TEZ and TTC with DD injection (left). The corresponding correlation of TTC with the contrast defect size following DMB injection is shown (right).

DMB injections consistently produced a contrast defect within the infarct zone at 0.5 MI immediately following bolus injection in all but two rats, who subsequently were found to have no or smaller-sized infarctions (S1 Table). In all rats that received DMB injections, myocardial contrast in the normal zones had washed out by two minutes (Fig 1; Panels B and D). The myocardial contrast defect size obtained within the first two minutes following bolus injection when imaging at 0.5 MI correlated with infarct size by TTC staining (r = 0.81; p<0.001; Fig 5). When the MI was transiently increased to 1.5 at two minutes or longer following the injection, no additional myocardial contrast was produced within the normal or infarct zones in all eight rats where it was tested.

In comparison, DD injections did not produce myocardial enhancement following bolus injection at the 0.5 MI in any animal and just trace amounts of LV cavity contrast (Fig 3A). When the MI was transiently increased over a five second time period to 1.5 MI at two minutes after injection, there was contrast enhancement with contrast pulse sequencing within the left ventricular cavity and normal myocardial segments during the high MI period. The TEZ was typically observed for a period of one to three frames within the hypokinetic zone (Fig 2 and Fig 3). After the third high MI frame, there was a reduction in contrast within the TEZ (Fig 2 and Fig 3). The size of the TEZ area correlated with infarct size by TTC staining (r = 0.94, p<0.001), and the correlation of TEZ size with TTC was similar to that seen with a contrast defect immediately following DMB injections (Fig 5). If the MI was transiently increased to 1.5 later than six minutes after injection, there was minimal evidence of droplet activation.

**Fig 6:**
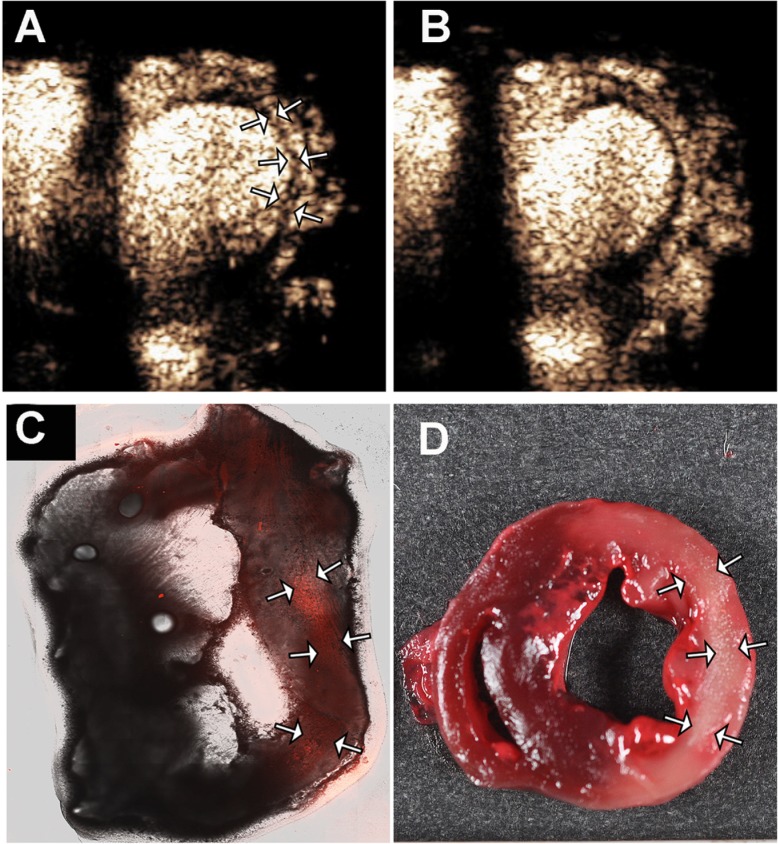
Transient zone of contrast enhancement (arrows, Panel A), in the first frames after increasing MI to 1.5 in a rat with an extensive lateral wall infarction by TTC (panel D). Note that after five high MI impulses the contrast disappears from the infarct zone (Panel B). Panel C demonstrates the fluorescent microscopy images of enhancement within the infarct zone (white arrows) from DiL-fluorophore labelled droplets given 10 minutes prior to sacrifice.

Fluorescent staining was feasible in 11 of 14 rats. In 3 animals (rats 6, 13, and 14 in S1 Table), the slices for the staining technique were not adequately prepared for microscopy. In the remaining 11 animals, staining was not seen within any normal functioning myocardial segment. The spatial extent of fluorescent staining corresponded to the spatial location of the infarct zone by TTC (Fig 6; S1 Table). There was also a correlation between the size of the TEZ and fluorescent area, with a correlation coefficient of 0.67 (p = 0.03).

There was slight injection to injection variability in TEZ measurements with repeated measurements (mean difference in the six rats tested was 0.60 mm^2^; 95% CI for the mean of the difference in repeated measurements 1.30 mm^2^). The correlation between the measurements of TEZ following repeated injections was good (r = 0.94; p<0.001).

### Porcine studies

**Fig 7:**
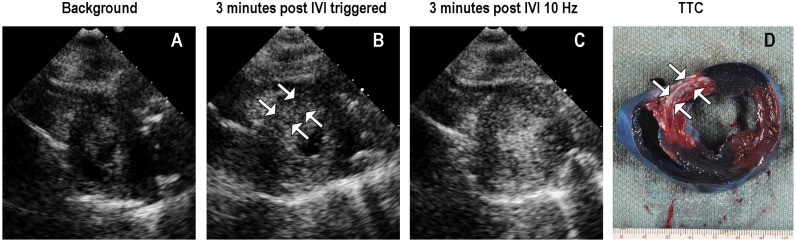
Selective infarct zone enhancement in a pig at 48 hours post ischemia/ reperfusion. Background signal intensity is displayed in Panel A. The selective enhancement within the infarct zone (white arrows Panel B) was observed with triggered 1.0 MI imaging. When increasing the frame rate to 10 Hz at 1.0 MI, there was droplet activation and imaging in both cavity and normal myocardium as well (Panel C). The corresponding TTC stain demonstrating the infarct zone is displayed in Panel D.

In an attempt to selectively activate and image droplets within the infarct zone and avoid attenuation from left ventricular cavity activation, the imaging modality was altered in the large animal model to single pulse harmonic imaging (1.3 or1.7 MHz transmit, 3.4 MHz receive) at slow frame rates, with attempts to activate and image at two and four minutes following IVI. Fig 7 displays an example of how selective activation and imaging of the droplets within the infarct zone was possible by reducing the frame rate to triggered end systolic imaging at an MI of 1.0 or 1.3. This figure also demonstrates that faster frame rates at this MI resulted in enhancement within normal myocardial segments as well as the left ventricular cavity. Furthermore, the duration of myocardial contrast enhancement within the infarct zone was longer at the higher MI settings than when using the multi-pulse setting in rats.

**Fig 8:**
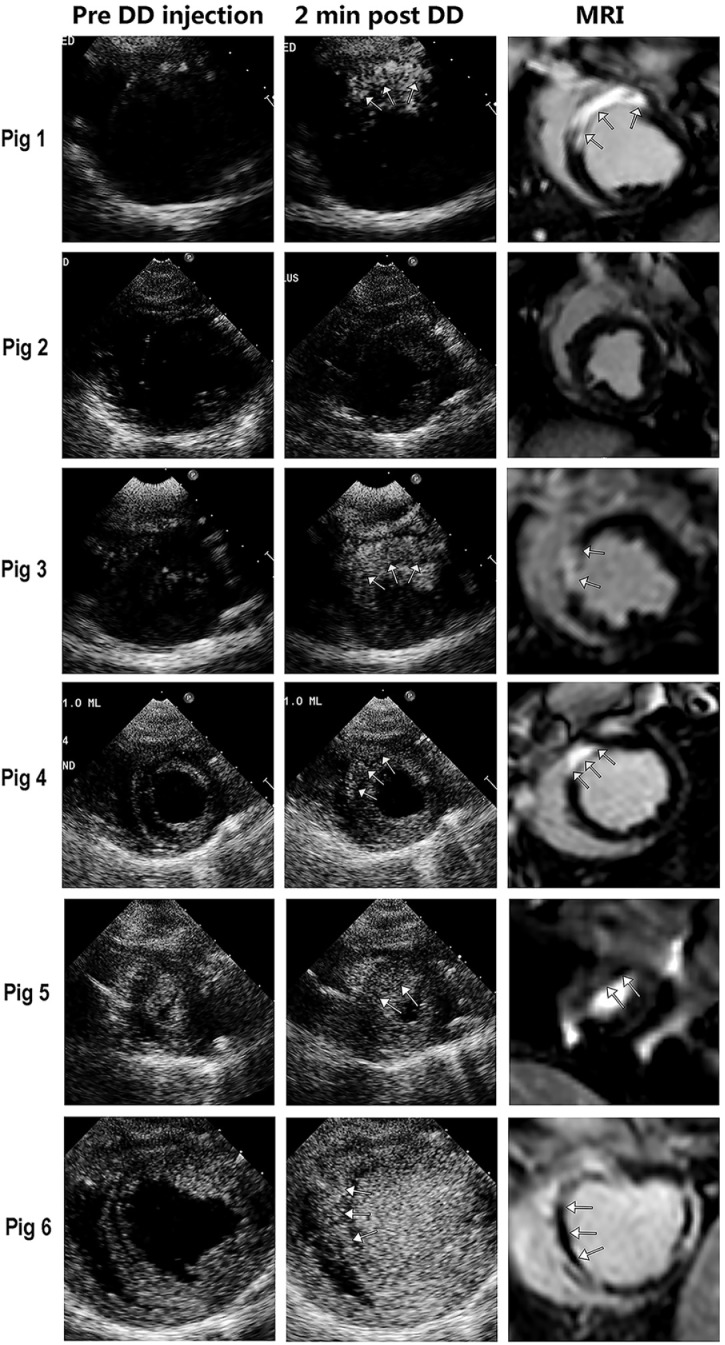
Enhancement within the anteroseptal scar zone with either ultraharmonic high MI imaging (Pigs 1 and 3) and 1.7 MHz harmonic high MI imaging (Pig 4–6) at over 2 minutes post IVI of 1.0 ml of DD. Pig 2 did not have an infarction following the 90 minute occlusion; pig 5 had a subendocardial infarction near the apex, and the other four pigs (1, 3, 4, and 6) had transmural infarctions. In Pig 5 the infarct by DE-MRI was only in an apical short axis segment, and the enhancement was confined to a subendocardial apical segment (arrows). In Pig 6, the infarct was large and accompanied by a microvascular obstruction that appears as a dark area within the central portion of the entire scar (arrows). The enhanced zone with harmonic or ultraharmonic imaging correlated closely with infarct zone location on CMRI, and with TTC measurements of infarct size. See text for details.

Transmural infarcts were observed in four of the six pig studies by DE-MRI and TTC staining (Fig 8 displays the images for all six pigs). At four minutes post 1.0 ml IVI of DD, slow frame rate triggered end systolic imaging at higher MIs (1.0–1.3) produced selective contrast enhancement within the infarct zone when using either ultraharmonic (1.3 MHz transmit, 3.4 MHz receive) or harmonic imaging (1.7 MHz transmit, 3.4 MHz receive), and minimal cavity contrast The 1.0 MI produced optimal selective activation of the infarct zone. There was less far field attenuation with the 1.7/3.4 MHz setting. Correlation of the selectively enhanced zone at 48 hours with infarct area size by TTC was good (r = 0.90, p<0.05).

## Discussion

The main findings of this study are that acoustically activated DD formulated from DMB have significantly different distribution kinetics in blood following IVI, which alters their clinical potential. DMB function as pure intravascular tracers, and can delineate an ischemic or infarcted zone as a contrast defect in the few seconds following a small bolus injection of the contrast agent. DD, on the other hand, remain in the circulation for extended periods of time following IVI, and appear to accumulate within regions of altered endothelial function that exist in the acute infarct or ischemia setting [9, 10], resulting in droplet accumulation within the risk area. Secondly, the droplets within this zone can be activated and imaged transiently with high MI impulses that utilize a non-linear pulse sequence schemes several minutes following injection, resulting in delayed detection and quantification of infarct size. Thirdly, we demonstrated in pigs that selective enhancement of the droplets within the infarct zone is feasible using a single pulse low frame rate activation and harmonic imaging sequence.

At low MIs (0.5 MI) following bolus injections of DD in the rodent models, there was no activation or contrast enhancement within the LV cavity or myocardium, while a similar concentration of intravenous DMB at this same MI produced myocardial contrast enhancement following bolus passage that was no longer evident at two minutes following IVI. This indicates rapid clearance of DMB from the systemic circulation. Furthermore, brief high MI impulses at two minutes post IVI did not produce any evidence of retained microbubbles within the risk area. DD, on the other hand, appear to persist for at least four minutes following IVI within both ischemia/reperfused zones and normal zones. Since the droplet shell composition was not different than the microbubble, the most likely explanation is the gas escapes from the microbubbles because of high surface tension, while the liquid fluorocarbon within droplets is at a lower surface tension and remained within the shell until either activation or shell metabolism. Their persistence within the infarct zone may indicate either increased adherence to leukocytes adherent to venular endothelium (which was not seen with DMB) or accumulation within the interstitial border zones known to exist in the infarction zones [17].

The timing of droplet activation and imaging within the infarct zone was a critical factor for determining the infarct zone location and size in the rat models of infarction. As the MI was increased to 1.5 with a multi-pulse sequence scheme at 2–4 minutes post IVI, there was cavity and normal myocardial enhancement, as well as transient enhancement within the infarct zone. The transient enhancement in the infarct zone can be explained by droplets that had accumulated within the infarct zone, but could not replenish in between high MI frames. Because the multi-pulse scheme (Contrast Pulse Sequencing) has a longer pulse sequence than standard imaging sequences [18], the longer exposure may account for the activation and imaging of faster moving droplets within the left ventricular cavity and normal microcirculation. Furthermore, the contrast pulse sequencing scheme is optimized for low MI imaging, and thus requires a reduction in gain and signal to noise ratio to reduce harmonic tissue signals.

This is why we modified the pulse sequence scheme for the pig studies, utilizing a shorter pulse sequence (harmonic single pulse), slightly lower MI (1.0), and a slow frame rate (end systolic triggered) for imaging and activation. A higher MI has consistently been required for activation and imaging of DD in normal myocardium with transthoracic imaging [7, 8], and higher peak negative pressures have been required for this phase change to occur outside the heart with higher molecular weight fluorocarbon droplets [19–22]. In this study, we discovered that it is possible to selectively activate droplets based on their velocity within the microcirculation. It was assumed that droplets retained within the infarct zone would have very slow or random motion (depending on their exact location), and short pulse harmonic sequences would be able to selectively activate these at a lower MI threshold due to the more prolonged exposure of droplets within the scar zone to the sinusoidal perturbations of ultrasound that enhance vaporization [6]. This was further improved by allowing for droplet accumulation to occur within the infarct zone prior to attempting activation, and reducing the number of droplets within the left ventricular cavity and normal microcirculation at two to four minutes post IVI.

### Cardiovascular clinical application

Although microbubble contrast agents have always been considered pure intravascular tracers, the re-formulation of them into droplets changes their characteristics and clinical utility. First, they could potentially be imaged at a high MI immediately after injection, allowing them to be reconverted back to microbubbles when they reach the left ventricular cavity and assess myocardial perfusion as has previously been demonstrated [7]. Secondly, because of their longer persistence within the circulation, they could be reactivated at several minutes following IVI to identify areas of altered endothelial function as was demonstrated in the current study. The potential for infarct zone quantification in this setting would be similar to DE-MRI [23–26], and may be applicable to other settings where altered microvascular permeability and endothelial dysfunction from scarring or inflammation occur, such as myocarditis, infiltrative diseases, transplant rejection, or hypertrophic cardiomyopathy.

### Limitations

The timing of the droplet activation chosen for this study was set to two or four minutes post IVI, mainly because at this time microbubble bolus injections were no longer producing myocardial contrast and were clearing the blood pool. It is unclear how long after IVI one may still be able to produce sufficient droplet activation that would permit enhancement exclusively within an infarct zone. The longer the time interval, the more likely there would be less interference from cavity droplet activation, and “interfering” myocardial contrast from activated droplets in normal zones. Shorter harmonic pulses significantly improve this selective enhancement, but activation and imaging sequences need to be optimized further and tested in inferior infarctions, where attenuation of harmonic impulses may occur. As can be seen in S1 Table, fluorescent intensity area was spatially located with the infarct location, but the correlation with TEZ area was weaker, indicating not all droplets within the infarct zone were being activated. Furthermore, we detected fluorescence within the infarct zone at 10 minutes following IVI, but were only able to demonstrate acoustic activation up to six minutes following injection. Although the transducers and pulse sequence schemes used for the current study were effective in droplet activation, more complex activation sequences may be required to obtain uniform droplet activation [27–28].

High background signal intensities in regions outside of the myocardium (e.g. pericardial reflections) can be seen prior to droplet injection due to non-linear signal generation as a function of distance from the transducer (Figs 7 and 8). However, the gain was reduced such that minimal signal was noted from the myocardium prior to droplet injection, even at the high mechanical index. Although the triggered end systolic technique appeared to prevent activation of cavity and normal myocardial droplets, slow flow in more apical short axis segments (Pig 5 in Fig 8) may result in activation in non-infarcted segments. To reduce the impact of this on detecting delayed enhancement, future development may require background subtraction techniques similar to what has been used for high mechanical index contrast microbubble imaging [10].

## Conclusion

DD produced from compressed commercially available perfluoropropane microbubbles accumulate within acute myocardial infarct zones, and serve as a method of infarct enhancement imaging with conventional echocardiographic pulse sequence schemes at prolonged time periods following intravenous injection. Selective activation of the infarct zone is possible with IV DD and a harmonic imaging sequence, similar to what is observed with DE-MRI. Although this would be considered an off-label use for the Definity ultrasound enhancing agent, the potential for translation into clinical practice is high, as the pulse sequence schemes which activate the droplets within the infarct zone are commercially available, and the droplet formulation process appears to be safe, bedside technique.

## Supporting information

S1 File Data files for each rat. (XLSX)

S2 File Data files for each pig. (XLSX)

S3 File ARRIVE Guidelines Checklist. (PDF)

S1 Table Individual measurements of the contrast defect. (DOCX)

## Abbreviations

DMB: Definity Microbubbles
DD: Definity Nanodroplets
DiI: Dioctadecyltetramethylindocarbocyanine
IVI: Intravenous Injection
LAD: Left Anterior Descending Artery
LV: Left Ventricular
MB: Microbubbles
MI: Mechanical Index
TTC: Triphenyltetrazolium Chloride
