# Supplementary material for: Selective infarct zone imaging with intravenous acoustically activated droplets
Source: PLoS One. 2018 Dec 14;13(12):e0207486. doi: 10.1371/journal.pone.0207486 (PMC6294612; doi:10.1371/journal.pone.0207486)
Supplement: S1 Table — (DOCX) [file pone.0207486.s004.docx]

**S1 Table.** Individual measurements of the contrast defect determined with intravenous DMB injections versus the TEZ measurements at 2-4 minutes after IV DD injections. The corresponding fluorescent areas and TTC measurements from the post mortem slices at the equivalent short axis plane are displayed. Fluorescent images were not possible for rats 6, 13, and 14. Microbubble injection was not performed for Rat 12.

| Rat | DMB  Defect (mm^2^) | DD TEZ  (mm^2^) | Fluorescent  Area (mm^2^) | TTC  (mm^2^) |
| --- | --- | --- | --- | --- |
| 1 | 8.6 | 7.2 | 8.0 | 7.6 |
| 2 | 9.4 | 9.4 | 7.6 | 11.6 |
| 3 | 7.1 | 7.9 | 8.0 | 8.0 |
| 4 | 10.1 | 10.5 | 10.1 | 11.7 |
| 5 | 0 | 3.7 | 4.7 | 4.3 |
| 6 | 8.4 | 8.5 | * | 9.0 |
| 7 | 3.3 | 6.3 | 6.1 | 6.8 |
| 8 | 6.0 | 6.5 | 15.8 | 6.3 |
| 9 | 0 | 0 | 1.0 | 0 |
| 10 | 0 | 5.9 | 7.3 | 6.3 |
| 11 | 5.3 | 4.8 | 4.5 | 4.1 |
| 12 | - | 8.0 | 9.2 | 7.9 |
| 13 | 6.1 | 6.1 | * | 9.0 |
| 14 | 7.5 | 7.8 | * | 10.7 |
| Mean ± SD | 5.5 ± 3.6 | 6.6 ± 2.5 | 7.4 ± 3.6 | - 1. ± 3.2 |

*****Fluorescent imaging data not available in these rats.
